# Vaginitis and risk of sexually transmitted infections: results of a multi-center U.S. clinical study using STI nucleic acid amplification testing

**DOI:** 10.1128/jcm.00816-24

**Published:** 2024-08-14

**Authors:** Jane R. Schwebke, Paul Nyirjesy, Melissa Dsouza, Damon Getman

**Affiliations:** 1 University of Alabama at Birmingham, Birmingham, Alabama, USA; 2 Sidney Kimmel Medical College, Thomas Jefferson University, Philadelphia, Pennsylvania, USA; 3 Hologic, Inc., San Diego, California, USA; Johns Hopkins University, Baltimore, Maryland, USA

**Keywords:** bacterial vaginosis, vulvovaginal candidiasis, sexually transmitted infection, Nugent score, Amsel criteria, *Mycoplasma genitalium*, *Trichomonas vaginalis*

## Abstract

Significant increases in rates of sexually transmitted infections (STIs) caused by *Trichomonas vaginalis* (TV), *Chlamydia trachomatis* (CT), *Neisseria gonorrhoeae* (NG), and *Mycoplasma genitalium* (MG) are occurring in the United States. We present results of a U.S. study examining the intersection of STIs and vaginitis. Among 1,051 women with diagnoses for the presence or absence of bacterial vaginosis (BV) and/or symptomatic vulvovaginal candidiasis (VVC), 195 (18.5%) had one or more STIs, including 101 (9.6%) with TV, 24 (2.3%) with CT, 9 (0.8%) with NG, and 93 (8.8%) with MG. STI prevalence in BV-positive women was 26.3% (136/518), significantly higher than STI prevalence of 12.5% (59/474) in BV-negative women (*P* < 0.0002). Unlike infections with CT or NG, solo infections of MG or TV were each significantly associated with a diagnosis of BV-positive/VVC-negative (OR 3.0751; 95% CI 1.5797–5.9858, *P* = 0.0113, and OR 2.873; 95% CI 1.5687–5.2619, *P* = 0.0017, respectively) and with mixed infections containing MG and TV (OR 3.4886; 95% CI 1.8901–6.439, *P* = 0.0042, and OR 3.1858; 95% CI 1.809–5.6103, *P* = 0.0014, respectively). TV and MG infection rates were higher in all Nugent score (NS) categories than CT and NG infection rates; however, both STIs had similar comparative prevalence ratios to CT in NS 6–10 vs NS 0–5 (CT: 3.06% vs 1.4%, 2.2-fold; MG: 10.7% vs 6.1%, 1.8-fold; TV: 14.5% vs 7.0%, 2.1-fold). NG prevalence was relatively invariant by the NS category. These results highlight the complexity of associations of STIs with two major causes of vaginitis and underscore the importance of STI testing in women seeking care for abnormal vaginal discharge and inflammation.

**IMPORTANCE:**

This study reports high rates for sexually transmitted infections (STIs) in women seeking care for symptoms of vaginitis and bacterial vaginosis, revealing highly complex associations of STIs with two of the major causes of vaginal dysbiosis. These results underscore the importance of STI testing in women seeking care for abnormal vaginal discharge and inflammation.

## INTRODUCTION

Sexually transmitted infections (STIs) caused by *Trichomonas vaginalis* (TV), *Chlamydia trachomatis* (CT), *Neisseria gonorrhoeae* (NG), and *Mycoplasma genitalium* (MG) are increasing in the United States even though the United States has the highest health care consumption expenditures per capita compared to other high-income nations (1, 2). Reasons for the elevated infection rates are multifactorial, including funding decreases for specialty STI clinics exacerbating barriers to accessing care, stigmas associated with seeking care for STI screening or treatment, and the fact that many cases of infection are asymptomatic (3, 4). Complications of untreated STIs in women include infertility, preterm birth, congenital infections, and increased risk for HIV infection (5). Additional interventions need to be instituted to help control rates of STIs.

Vaginitis, defined as inflammation or infection of the vagina and vaginal epithelium, is associated with a spectrum of symptoms, including vulvovaginal itching, burning, irritation, dyspareunia, “fishy” vaginal odor, and abnormal vaginal discharge (6). The syndrome is the most common reason for women to seek medical care worldwide, and most women experience an episode of vaginitis at least once in their lifetimes (7–10). Although not classified as notifiable diseases, the vaginal infections or microbial dysbioses leading to symptoms of vaginitis, including bacterial vaginosis (BV), trichomoniasis, and vulvovaginal candidiasis (VVC), have an estimated prevalence of over 20 million women in the United States for BV (11) and 3.7 million cases of trichomoniasis (12). An estimated 75% of women will have at least one lifetime episode of VVC due to overgrowth of *Candida* species (10). Trichomoniasis, caused by infection with the protozoan *Trichomonas vaginalis* (TV), is well established as an STI as well as a cause of vaginitis and is the most common non-viral STI (13–15).

Numerous previous studies describe an increased rate of STIs among women with vaginitis (16–20). More recent data generated using state-of-the-art nucleic acid amplification tests (NAATs) are available from studies investigating vaginitis as a biological risk factor for STIs (21, 22). To expand on these previous analyses, in this study, we present the results of STI NAAT testing examining the intersection of vaginitis and the STIs CT, NG, TV, and MG in women seeking care at a variety of clinical practice types in the United States

## MATERIALS AND METHODS

### Study design

Remnant specimens obtained from a previous prospective multi-center diagnostic accuracy cross-sectional study (23) conducted to validate the clinical performance of two FDA-cleared nucleic acid amplification tests for BV and VVC were used for investigating the prevalence and distribution of sexually transmitted organisms. Details on the enrollment and consent procedures for participants in this study are described previously. Participants were compensated for study participation.

### Study population

Persons at least 14 years of age with symptoms of vaginitis (e.g., abnormal vaginal discharge, vaginal odor, genital itching or irritation, pain/discomfort during sexual intercourse or urination, edema, or erythema) were eligible for enrollment. Enrollment occurred at 21 U.S. sites, consisting of clinical research centers and emergency medicine, family planning, public health, STI, and family medicine/obstetric-gynecologic clinics, between June and October 2018. For each subject, the collection site provided subject demographic and clinical data, including clinician’s diagnosis, subject-reported date of birth, sex, ethnicity, race, symptoms of STIs, pregnancy status, menstrual status, recent unprotected sexual intercourse (i.e., within 24 h), HIV diagnosis, history of recurrent symptoms of vaginitis within 12 months, and use of feminine products within 4 weeks. Of 1,168 patient specimens available for STI NAAT analysis, 64 were missing a BV diagnosis result and 53 had insufficient specimen volume or invalid or equivocal NAAT test result for CT, NG, or MG, yielding complete results from 1,051 persons available for analysis.

### Sample collection

Vaginal swab samples for use in molecular testing were collected in the clinic from each patient during routine clinical visits using Aptima Multitest swabs (Hologic, Inc., San Diego, CA). Also collected were one swab for *Candida* spp. culture (BD BBL CultureSwab EZ; Becton, Dickinson and Company; Sparks, MD) and one cotton swab each for Nugent score/Amsel criteria and *T. vaginalis* culture. The Gram slide was left to air dry before being sent to the reference laboratory for fixation and staining. The wet mount slide was evaluated for modified Amsel criteria by the clinician within 20 min from the time of collection.

### Laboratory testing

For diagnosis of BV, the reference method was comprised of a consensus Nugent score and modified Amsel criteria if necessary (described below). For each subject, a single clinician-collected vaginal swab was first smeared on a glass microscope slide to prepare the Nugent scoring slide and then used to complete Amsel evaluation. The slide was then Gram stained and assigned a Nugent score, as described previously (24). Each Gram-stained slide was independently reviewed by three different reviewers at a single reference laboratory, blinded to each other’s interpretations. Agreement on BV interpretations (positive, negative, intermediate) by at least two reviewers constituted consensus, and the Nugent interpretation was final. Disagreement across all three reviewers was resolved via panel review of the same slide at a multi-headed microscope. Slides with a consensus Nugent interpretation of intermediate were resolved using modified Amsel criteria (≥20% clue cells together with either vaginal fluid pH greater than 4.5 or a positive whiff test (potassium hydroxide test on the swab) to determine BV status (25).

For diagnosis of VVC, the reference method was comprised of yeast culture. For each subject, a single vaginal swab was used to inoculate two different culture media at a single reference laboratory: Sabouraud Dextrose Agar and CHROMagar Candida (CHROMagar, Paris, France). The growth level on both media after 48 h was reported as follows: no colony, 1+, 2+, 3+, and 4+, with *n*+ representing the number of quadrants showing *Candida* growth. Subjects with a positive culture result with either medium were categorized as positive for VVC. For trichomoniasis, the reference method was comprised of the combined results of an FDA-cleared NAAT for *T. vaginalis* and culture, as described previously (23).

Clinician-collected vaginal swab specimens that had been stored at −70°C were tested for the sexually transmitted organisms *Neisseria gonorrhoeae* (NG) and *Chlamydia trachomatis* (CT), and *Mycoplasma genitalium* (MG), using FDA-cleared transcription-mediated amplification NAATs (Aptima Combo 2 and Aptima *Mycoplasma genitalium*, respectively) for the detection of ribosomal RNA from each organism. All NAAT testing was performed at one site on the automated Panther system instrument using assay-specific software for results interpretation.

### Statistical methods

Demographic characteristics were evaluated based on the vaginosis/vaginitis laboratory diagnosis. Relative risks (RR) were also computed along with the Wald confidence intervals (CI) for these estimates. Adjusted odds ratios (aOR) were obtained from multivariable logistic regression models along with the corresponding Wald *CIs*. Results were considered significant at the level of *α* ≤ 0.05. Samples with inconclusive reference results and samples with invalid or missing investigational assay results were excluded from the analyses. Analyses were performed with SAS software (version 9.4; SAS Institute Inc, Cary, NC).

## RESULTS

Specimens from 1,051 women were tested for the presence of *N. gonorrhoeae*, *C. trachomatis*, *M. genitalium*, and *T. vaginalis* by NAAT, and infection status was then compared to laboratory-based consensus Gram stain diagnosis for bacterial vaginosis (BV) or culture for vulvovaginal candidiasis (VVC) diagnosis from overgrowth of *Candida* species.

Table 1 shows the distribution of reference laboratory diagnosis (BV or VVC) categories by age range, self-reported race/ethnicity, and reported symptoms. For all women evaluated, 385 (36.6%) had a diagnosis of only BV (BV+/VVC−), 179 (17.0%) were diagnosed with VVC only (BV−/VVC+), 138 (13.1%) were BV+/VVC+, and 349 (33.2%) were BV−/VVC−. The prevalence of each vaginal disorder diagnosis category was largely consistent across all age range groups from 14 to 77 years although women in the 61–77 years age group represented only 1.8% of those evaluated, and some categories (e.g., BV+/VVC+) in that age range had few to no persons enrolled. Black women had the highest prevalence of BV diagnosis, for both BV+/VVC− and BV+/VVC+ categories, compared to other races, and the lowest prevalence of BV−/VVC+ diagnosis. Women with a diagnosis of BV+/VVC− or BV+/VVC+ had significantly elevated relative risks for abnormal discharge (RR 2.02; 95% CI 1.59–2.57, *P* < 0.0001 and RR 3.38; 95% CI 1.94–5.90, *P* < 0.0001, respectively), abnormal odor (RR 2.64; 95% CI 2.27–3.07, *P* < 0.0001 and RR 2.00; 95% CI 1.46–2.75, *P* < 0.0001, respectively), and positive modified Amsel criteria (RR 7.61; 95% CI 5.84–9.89, *P* < 0.0001 and RR 3.69; 95% CI 2.54–5.36, *P* < 0.0001, respectively). Women with a diagnosis of BV−/VVC+ or BV−/VVC− had significantly elevated relative risks only for genital symptoms of itch, irritation, burning, or soreness (RR 2.33; 95% CI 1.69–3.22, *P* < 0.0001 and RR 1.22; 95% CI 1.02–1.46, *P* < 0.03, respectively).

**TABLE 1:** Demographic characteristics and laboratory diagnosis of women with symptoms of vaginitis^*c*^^,^^*d*^

		Vaginosis/vaginitis laboratory diagnosis			
Subject characteristic	*N* (%)	BV+/VVC−	BV+/VVC+	BV−/VVC+	BV−/VVC−
Age 14–77	1,051 (100)	385 (36.6)	138 (13.1)	179 (17.0)	349 (33.2)
14–25	242 (23.0)	94 (38.8)	42 (17.4)	40 (16.5)	66 (27.3)
26–30	203 (19.3)	73 (36.0)	27 (13.3)	36 (17.7)	67 (33.0)
31–40	337 (32.1)	130 (38.6)	39 (11.6)	54 (16.0)	114 (33.8)
41–50	161 (15.3)	57 (35.4)	16 (9.9)	30 (18.6)	58 (36.0)
51–60	89 (8.5)	29 (32.6)	9 (10.1)	19 (21.3)	32 (36.0)
61–77	19 (1.8)	2 (10.5)	0	5 (26.3)	12 (63.2)
Race					
Asian	52 (4.9)	14 (26.9)	5 (9.6)	10 (19.2)	23 (44.2)
Black/African American	520 (49.5)	243 (46.7)	84 (16.2)	75 (14.4)	118 (22.7)
White (Hispanic/Latina)	179 (17.0)	57 (31.8)	24 (13.4)	32 (17.9)	66 (36.9)
White (Not Hispanic/Latina)	252 (24.0)	55 (21.8)	16 (6.3)	55 (21.8)	126 (50.0)
Other	48 (4.6)	16 (33.3)	4 (8.3)	12 (25.0)	16 (33.3)
Abnormal discharge					
Yes	769 (73.2)	326 (42.4)	120 (15.6)	134 (17.4)	189 (24.6)
No	282 (26.8)	59 (20.9)	13 (4.6)	50 (17.7)	160 (56.7)
RR (95% CI)		**2.0262** **(1.5917–2.5795) *P* = <**0.0001	**3.3850** **(1.9417–5.9011) *P* = <**0.0001	0.9828 (0.7319–1.3197) *P* = 0.9081	**0.4332** **(0.3690–0.5085) *P* = <**0.0001
Abnormal odor					
Yes	312 (29.7)	203 (65.1)	61 (19.6)	12 (3.8)	36 (11.5)
No	739 (70.3)	182 (24.6)	72 (9.7)	172 (23.3)	313 (42.4)
RR (95% CI)		**2.6419** **(2.2737–3.0696) *****P*** **= <0.0001**	**2.0067** **(1.4654–2.7480) *****P*** **= <0.0001**	**0.1653** **(0.0934–0.2922) *****P*** **= <0.0001**	**0.2724** **(0.1981–0.3746) *****P*** **= <0.0001**
Genital symptoms (itch/irritation/burning/soreness)					
Yes	622 (59.2)	184 (29.6)	73 (11.7)	142 (22.8)	223 (35.9)
No	429 (40.8)	201 (46.9)	60 (14.0)	42 (9.8)	126 (29.4)
RR (95% CI)		**0.6314** **(0.5393–0.7392) *P* = <0.0001**	0.8391 (0.6102–1.1540) *P* = 0.2807	**2.3319** **(1.6907–3.2162) *P* = <0.0001**	**1.2207** **(1.0191–1.4622) *P* = 0.0304**
Modified Amsel^*a*^					
Positive^*b*^	473 (45.1)	331 (70.0)	100 (21.1)	13 (2.7)	29 (6.1)
Negative	576 (54.9)	53 (9.2)	33 (5.7)	171 (29.7)	319 (55.4)
RR (95% CI)		**7.6052** **(5.8451–9.8955) *****P*** **= <0.0001**	**3.6902** **(2.5382–5.3649) *****P*** **= <0.0001**	**0.0926** **(0.0534–0.1606) *****P*** **= <0.0001**	**0.1107** **(0.0772–0.1587) *****P*** **= <0.0001**

^*a*^ Presence of ≥20% Clue cells, pH >4.5, positive whiff test.

^*b*^ Two subjects had unknown Amsel results.

^*c*^ Outcome variables are the subject characteristics; predictors are vaginitis/vaginosis laboratory diagnosis. A separate variable was created for each predictor variable, for BV+VVC+: Yes = all subjects with positive results for BV and VCC, No = all other subjects.

^*d*^ Bold values indicate significant comparisons. BV, bacterial vaginosis; VVC, vulvovaginal candidiasis, RR, relative risk.

Table 2 lists the prevalence of STI diagnoses by age range and self-reported race/ethnicity demographic category, as well as associations of each STI with reported genital symptoms and modified Amsel criteria. For all women (*n* = 1,051), 195 (18.5%) had one or more STIs, including 101 (9.6%) with TV, 24 (2.3%) with CT, 9 (0.8%) with NG, and 93 (8.8%) with MG. For adolescent and adult women ages 14–25 years, MG had the highest prevalence (15.3%), followed by TV (6.2%), CT (5.4%), and NG (1.2%). Women in older age brackets, in general, had increasingly lower STI prevalence, except for TV where prevalence in women ages 41–60 years (13.2%) was nearly double that of TV prevalence in women ages 14–30 years (6.9%), and for MG, where some infections were seen in women ages 51–70. Women identifying as Black/African American had the highest overall STI prevalence (128/520, 24.6%), followed by Other race/ethnicity (10/48, 20.8%), White/Hispanic Latina (28/179, 15.6%), Asian (5/52, 9.6%), and White/Not Hispanic Latina (24/252, 9.5%).

**TABLE 2:** Sexually transmitted infection prevalence and distribution among 1,051 women with symptoms of vaginitis/vaginosis^*b*^

Subject characteristic	No (%)	*T. vaginalis*	*C. trachomatis*	*N. gonorrhoeae*	*M. genitalium*	Any STI
Age 14–77	1,051 (100)	101 (9.6)	24 (2.3)	9 (0.8)	93 (8.8)	195 (18.5)
14–25	242 (23)	15 (6.2)	13 (5.4)	3 (1.2)	37 (15.3)	57 (23.6)
26–30	203 (19.3)	16 (7.9)	6 (2.9)	2 (0.9)	19 (9.4)	42 (20.7)
31–35	185 (17.6)	21 (11.4)	1 (0.5)	2 (1.1)	17 (9.2)	35 (18.9)
36–40	152 (14.5)	16 (10.5)	2 (1.3)	1 (0.7)	7 (4.6)	19 (12.5)
41–45	97 (9.2)	14 (15.7)	0 (0.0)	1 (1.0)	5 (5.1)	17 (17.5)
46–50	64 (6.1)	5 (7.8)	2 (3.1)	0 (0.0)	4 (6.2)	8 (12.5)
51–60	89 (8.5)	14 (15.7)	0 (0.0)	0 (0.0)	3 (3.4)	16 (18.0)
61–70	17 (16.2)	0 (0.0)	0 (0.0)	0 (0.0)	1 (5.9)	1 (5.9)
71–77	2 (0.2)	0 (0.0)	0 (0.0)	0 (0.0)	0 (0.0)	0 (0.0)
Race, ethnicity						
Asian	52 (4.9)	4 (7.7)	0 (0.0)	0 (0.0)	1 (1.9)	5 (9.6)
Black/African American	520 (49.5)	70 (13.5)	16 (3.1)	4 (0.8)	58 (11.1)	128 (24.6)
Other	48 (4.6)	3 (6.2)	0 (0.0)	0 (0.0)	9 (18.7)	10 (20.8)
White (Hispanic/Latina)	179 (17.0)	12 (6.7)	2 (1.1)	4 (2.2)	16 (8.9)	28 (15.6)
White (Not Hispanic/Latina)	252 (24.0)	12 (4.8)	6 (2.4)	1 (0.4)	9 (3.6)	24 (9.5)
Abnormal discharge	No	26 (25.7)	3 (12.5)	1 (11.1)	19 (20.4)	43 (22.1)
	Yes	75 (74.3)	21 (87.5)	8 (88.9)	74 (79.6)	152 (77.9)
		1.0578 (0.6918–1.6175) *P* = 0.7953	2.5670 (0.7716–8.5397) *P* = 0.1242	2.9337 (0.3686–23.3506) *P* = 0.3092	1.4282 (0.8791–2.3203) *P* = 0.1500	1.2963 (0.9509–1.7671) *P* = 0.1007
Abnormal odor	No	57 (56.4)	13 (54.2)	6 (66.7)	58 (62.4)	114 (58.5)
	Yes	44 (43.6)	11 (45.8)	3 (33.3)	35 (37.6)	81 (41.5)
		**1.8284 (1.2624–2.6481)*P* = 0.0014**	2.0042 (0.9078–4.4247) *P* = 0.0853	1.1843 (0.2981–4.7054) *P* = 0.8101	1.4293 (0.9599–2.1282) *P* = 0.0786	**1.6829 (1.3078–2.1657)*****P*** **= <0.0001**
Genital symptoms (itch/irritation/ burning/soreness)	No	41 (40.6)	11 (45.8)	1 (11.1)	51 (54.8)	93 (47.7)
	Yes	60 (59.4)	13 (54.2)	8 (88.9)	42 (45.2)	102 (52.3)
		1.0093 (0.6919–1.4724) *P* = 0.9615	0.8151 (0.3687–1.8023)*P* = 0.6136	5.5177 (0.6926–43.9552) *P* = 0.1067	**0.5680 (0.3848–0.8384) *P* = 0.0044**	**0.7565 (0.5876–0.9739)*P* = 0.0304**
Modified Amsel^*a*^	Negative	38 (38.0)	9 (37.5)	4 (44.4)	36 (38.7)	75 (38.7)
	Positive	62 (62.0)	15 (62.5)	5 (55.6)	57 (61.3)	119 (61.3)
		**1.9869 (1.3519–2.9201) *P* = 0.0005**	2.0296 (0.8962–4.5962)*P* = 0.0896	1.5222 (0.4111–5.6367) *P* = 0.5293	**1.9281 (1.2936–2.8739) *P* = 0.0013**	**1.9322 (1.4866–2.5112)*P* = <0.0001**

^*a*^ Clue cells, pH > 4.5, KOH whiff test.

^*b*^ Bold values indicate significant comparisons.

TV infection was significantly associated with abnormal odor (OR 1.8284; 95% CI 1.2624–2.6481, *P* = 0.0014) and positive modified Amsel criteria (OR 1.9869; 95% CI 1.3519–2.9201, *P* = 0.0005). MG infection was significantly associated with only positive modified Amsel criteria (OR 1.9821; 95% CI 1.2936–2.8739, *P* = 0.0013). CT and NG infections were not significantly associated with any reported symptom or modified Amsel criteria status.

**Fig 1:**
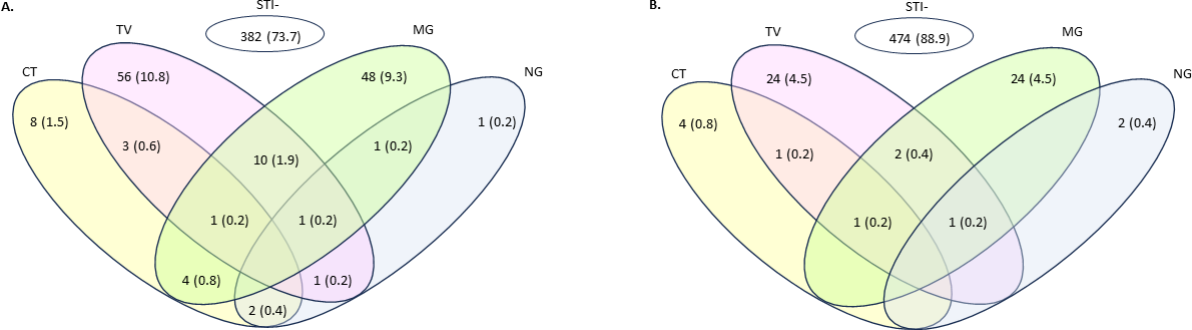
Distribution of sexually transmitted infections (*n* = 195, 18.5%) in 1,051 women with symptoms of vaginitis/vaginosis. (**A**) STI distribution in laboratory-diagnosed BV-positive women (*n* = 518) and (**B**) in BV-negative women (*n* = 533). Overall prevalence for each STI: *N. gonorrheoae* (NG), 0.9% (*n* = 9); *C. trachomatis* (CT), 2.3% (*n* = 24); *M. genitalium* (MG), 8.8% (*n* = 93); *T. vaginalis* (TV*)*, 9.6% (*n* = 101).

The distribution of single and multiple STIs by BV diagnosis status is shown in Fig. 1. Overall STI prevalence in BV-positive women was 26.3% (136/518), significantly higher than STI prevalence of 12.5% (59/474) in BV-negative women (*P* < 0.0002). Infections consisting of a single STI were predominant and slightly higher in BV-negative (91.5%, 54/59) than BV-positive (83%, 113/136) women, while BV-positive women had higher diversity of STI single organism and co-organism infection states (12 combinations) compared to BV-negative women (eight combinations).

Analysis of the association of STIs with BV and VVC diagnoses is shown in **Table 3**. Compared to women with a diagnosis of BV−/VVC−, neither NG solo infection nor NG mixed infection with another STI was significantly associated with any BV or VVC diagnosis. Combined mixed and solo infections with CT (Any CT+) were significantly associated only with a diagnosis of BV+/VVC− (OR 4.676; 95% CI 1.342–16.291, *P* = 0.0192), while solo CT infections (Only CT+) were not associated with any BV or VVC diagnosis. MG and TV solo infections were each significantly associated with a diagnosis of BV+/VVC− (OR 3.0751; 95% CI 1.5797–5.9858, *P* = 0.0113 and OR 2.873; 95% CI 1.5687–5.2619, *P* = 0.0017, respectively) as were mixed infections containing MG and TV and another STI (OR 3.4886; 95% CI 1.8901–6.439, *P* = 0.0042 and OR 3.1858; 95% CI 1.809–5.6103, *P* = 0.0014, respectively). Mixed infections containing TV and another STI were also associated with a diagnosis of BV+/VVC+ (OR 3.0565; 95% CI 1.524–6.1301, *P* = 0.0297).

**TABLE 3:** Association of sexually transmitted infections with laboratory-diagnosed bacterial vaginosis and vulvovaginal candidiasis^*a*^^,^^*b*^

		Laboratory diagnosis					
	BV−/VVC−	BV+/VVC−		BV−/VVC+		BV+/VVC+	
STI	(Reference)	No. (%)	OR (95% CI)	No. (%)	OR (95% CI)	No. (%)	OR (95% CI)
Any CT+	3 (0.9)	15 (3.9)	**4.6757 (1.342–16.2908) *P* = 0.0192**	3 (1.6)	1.9116 (0.382–9.5669) *P* = 0.7669	3 (2.3)	2.6615 (0.5304–13.3546) *P* = 0.7023
Any NG+	1 (0.3)	4 (1)	3.6535 (0.4064–32.8453) *P* = 0.6725	2 (1.1)	3.8242 (0.3445–42.4564) *P* = 0.6736	2 (1.5)	5.313 (0.4777–59.0859) *P* = 0.3455
Any MG+	14 (4)	49 (12.7)	**3.4886 (1.8901–6.439) *P* = 0.0042**	14 (7.6)	1.97 (0.9182–4.2268) *P* = 0.6666	16 (12)	3.2714 (1.5492–6.9079) *P* = 0.0712
Any TV+	17 (4.9)	54 (14)	**3.1858 (1.809–5.6103) *P* = 0.0014**	12 (6.5)	1.3624 (0.6362–2.9177) *P* = 0.1666	18 (13.5)	**3.0565 (1.524–6.1301) *P* = 0.0297**
Only CT+	3 (0.9)	8 (2.1)	2.4474 (0.6441–9.2992) *P* = 0.9445	1 (0.5)	0.6302 (0.0651–6.1018) *P* = 0.9662	0 (0)	NC
Only NG+	0 (0)	1 (0.3)	19492.3859 (0–8.9149705659564E128) *P* = 0.9422	2 (1.1)	82253.5846 (0–3.7494287003591E129) *P* = 0.9237	0 (0)	NC
Only MG+	12 (3.4)	38 (9.9)	**3.0751 (1.5797–5.9858) *P* = 0.0113**	12 (6.5)	1.9591 (0.862–4.4523) *P* = 0.946	10 (7.5)	2.2829 (0.962–5.4177) *P* = 0.5306
Only TV+	15 (4.3)	44 (11.4)	**2.873 (1.5687–5.2619) *P* = 0.0017**	9 (4.9)	1.1451 (0.4912–2.6695) *P* = 0.1953	12 (9)	2.2082 (1.0051–4.8512) *P* = 0.2442

^*a*^ Bold values indicate significant comparisons.

^*b*^ NC, not calculable; OR, odds ratio.

**Fig 2:**
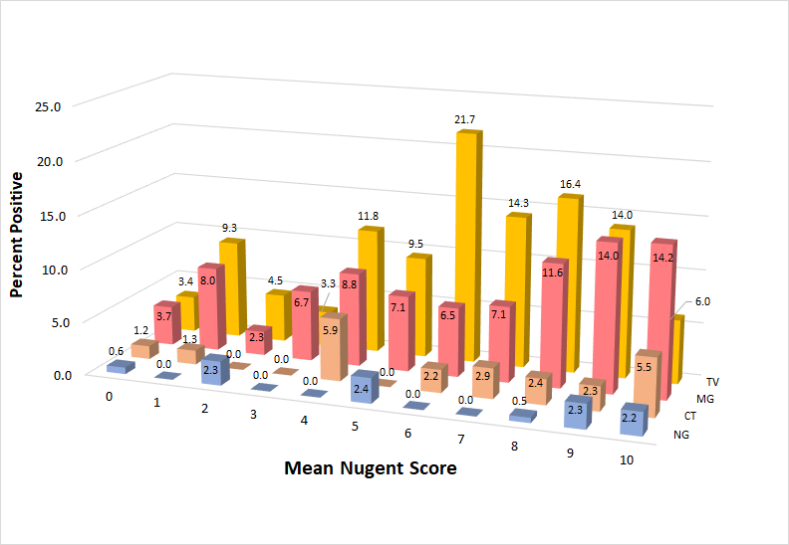
Prevalence of sexually transmitted infections by consensus Nugent score in women with symptoms of vaginitis/vaginosis.

Fig. 2 shows the prevalence of each STI categorized by consensus Nugent score (cNS). NG prevalence was relatively invariant with respect to cNS category, while the mean CT prevalence in cNS categories 6–10 (3.06%) was approximately twice (2.2-fold) that of mean CT prevalence in cNS categories 0–5 (1.4%). TV and MG infection prevalence values were higher in all cNS categories than CT and NG infection prevalence; however, both STIs had similar comparative prevalence ratios to CT in cNS 6–10 vs 0–5 (MG: 10.7% vs 6.1%, 1.8-fold; TV: 14.5% vs 7.0%, 2.1-fold).

A variety of factors including patient demographic status, STI infection status, age range, cNS, modified Amsel criteria status, and presence of vaginal symptoms were assessed in relation to the occurrence of patient-reported recurrent vaginitis or vaginosis in the past 12 months. As shown in Table 4, of these factors, only White Hispanic/Latina women had a modest but significant elevated risk of vaginitis recurrence in this time period (RR 1.18; 95% CI 1.0619–1.3188, *P* = 0.0023).

**TABLE 4:** Demographic factors and sexually transmitted infection prevalence in women with and without recurrent vaginitis/vaginosis^*a*^

		Vaginitis/vaginosis in last 12 months				
		No		Yes		
Factor	*N*	No.	%	No.	%	Relative risk 95% (CI)
Asian	52	21	40.4	31	59.6	1.0391 (0.8265–1.3065) *P* = 0.7425
Black/African American	518	207	40	311	60	1.0591 (0.9628–1.1650) *P* = 0.2380
White (Hispanic/Latina)	179	52	29.1	127	70.9	**1.1834 (1.0619–1.3188) *P* = 0.0023**
White (Not Hispanic/Latina)	251	105	41.8	146	58.2	0.9235 (0.8210–1.0388) *P* = 0.1848
Other	48	15	31.3	33	68.8	1.1179 (0.9181–1.3612) *P* = 0.2674
14–35	627	226	36	401	64	0.9174 (0.8306–1.0132) *P* = 0.0890
36–77	421	174	41.3	247	58.7	0.9174 (0.8306–1.0132) *P* = 0.0890
TV+	100	47	47	53	53	0.8444 (0.6976–1.0221) *P* = 0.0827
CT+	24	10	41.7	14	58.3	1.0614 (0.7543–1.4935) *P* = 0.7324
GC+	9	3	33.3	6	66.7	0.9269 (0.5825–1.4747) *P* = 0.7485
MG+	92	28	30.4	64	69.6	1.1388 (0.9857–1.3156) *P* = 0.0776
Any STI+	193	78	40.4	115	59.6	0.9558 (0.8415–1.0856) *P* = 0.4868
BV+	517	201	38.9	316	61.1	0.9776 (0.8888–1.0752) *P* = 0.6406
VVC+	316	117	37	199	63	1.0267 (0.9269–1.1372) *P* = 0.6139
Nugent 0–3	439	166	37.8	273	62.2	0.9902 (0.8994–1.0902) *P* = 0.8407
Nugent 4–6	98	36	36.7	62	63.3	0.9750 (0.8317–1.1430) *P* = 0.7550
Nugent 7–10	468	181	38.7	287	61.3	1.0149 (0.9221–1.1171) *P* = 0.7617
Modified Amsel criteria	472	182	38.6	290	61.4	0.9879 (0.8976–1.0872) *P* = 0.8028
Abnormal discharge	767	305	39.8	462	60.2	0.9100 (0.8222–1.0071) *P* = 0.0684
Abnormal odor	311	126	40.5	185	59.5	0.9469 (0.8506–1.0541) *P* = 0.3185
Genital itch	620	229	36.9	391	63.1	1.0503 (0.9522–1.1584) *P* = 0.3267

^*a*^ Bold values indicate significant comparisons.

## DISCUSSION

This study investigated the prevalence of sexually transmitted infections and association of STIs with symptomatology and clinical diagnosis of vaginitis in a large, diverse cohort of women in the United States seeking care for symptoms of vaginitis. To our knowledge, this is the most comprehensive study to date aimed at elucidating the association of STIs and this common gynecological complaint. In a study sample exhibiting typical characteristics (clinical signs, symptoms, and demographics) of women seeking care for gynecological symptoms of vagintis, we found complex patterns of STI infections and co-infections in women with and without a diagnosis of bacterial vaginosis. Overall, BV-positive women had a statistically significant twofold higher STI infection rate compared to women with a BV-negative diagnosis, and BV-positive women also had a 50% higher level of diversity of STI type co-infections than women with a negative BV diagnosis. Adjusting for co-diagnosis of vulvovaginal candidiasis, only *T. vaginalis* and *M. genitalium* were significantly associated with a diagnosis of BV and the presence of positive modified Amsel criteria, either as solo infections or as mixed infections with another STI. *C. trachomatis* and *N. gonorrhoeae* were not significantly associated with a BV diagnosis or signs and symptoms of BV, nor were any STIs significantly associated with a diagnosis of candidiasis.

Prior prospective studies have shown BV to be a significant risk factor for increases in prevalent and incident CT, NG, and TV infections (16–20). A recent meta-analysis (22) showed women with BV have a statistically significant increase in incident TV infection (aOR 1.87 (95% CI: 1.45–2.40) although the studies available for inclusion in that analysis used relatively insensitive wet mount and culture methods for diagnosis of TV infection, which may have led to an underestimate of the true rate of incident infections during the follow-up periods employed (26, 27). Use of sensitive NAAT methods for diagnosis of TV infection, as was done in this study, following a negative baseline diagnosis of TV infection may reveal that BV fosters higher risks for subsequent incident TV infection than previously reported.

Our study is the first to include MG in a comprehensive assessment of prevalent STIs in women using a laboratory-based consensus diagnosis of vaginitis or vaginosis. After adjusting for candidiasis and other STIs, we found MG infection was significantly associated with a diagnosis of BV. Previous studies have found similar significant associations for both prevalent and incident MG infections and BV. Using DNA PCR-based NAATs for MG detection, Nye et al. (28), Oakeshott et al. (29), and Shipitsyna et al. (18) found significant increases of prevalent MG infection in women with BV compared to BV-negative controls (OR 1.97, 95% CI 1.73–3.39; RR 2.73, 95% CI 1.73–4.30; and OR 2.60, 95% CI 1.11–6.15, respectively). Using a sensitive transcription-mediated amplification-based NAAT for MG rRNA detection, Lokken et al. (30) reported an odds ratio of prevalent MG infection in BV-positive vs -negative women of 3.76 (95% CI: 1.81–7.72) similar to the value we determined here (OR 3.07, 95% CI 1.58–5.99) using the same rRNA TMA-based NAAT for MG detection. Similar to incident TV infections, women with antecedent BV are reported to have significantly higher rates of incident MG infections compared to women with a historically negative BV status (29, 30).

Many studies have reported significant associations of MG infection with a diagnosis of cervicitis, pelvic inflammatory disease, and adverse pregnancy outcomes (5, 31); however, data describing similar associations of the organism with a diagnosis of non-BV vaginitis are less conclusive, with some studies showing significant correlation of MG infection with vaginal inflammation and others not (21, 32). Current CDC STI treatment guidelines support NAAT testing for MG in women with symptomatic cervicitis and pelvic inflammatory disease but is not recommended for asymptomatic women (10). Our data presented here show MG infection to be not associated with some of the symptoms of vaginitis (itch, irritation, burning, soreness) or with recurrence of symptoms of vaginitis or BV within 12 months of first diagnosis. However, as described above, our data do confirm and expand on previous reports showing MG infection alone is significantly associated with the signs, symptoms, and diagnosis of BV.

BV has long been considered a nuisance condition. This attitude is reflected in the lack of attention afforded to the condition by some care providers, leading to persistence in the employment of empiric diagnosis for vaginal disorders, encompassing methods which are often inaccurate and lead to incorrect therapy (33, 34). Just as STIs and HIV were once described as having “epidemiological synergy” (35), our study supports the concept that STIs and BV are intersecting clinical states with commonalities in disturbance of the vaginal microbiome that ultimately are inconsistent with optimal sexual and reproductive health. Given the rising rates of STIs in the United States and elsewhere, and the established adverse health outcomes associated with lack of diagnosis and treatment of them, every strategy should be pursued to decrease their prevalence.

To this end, the CDC recommends that all women diagnosed with BV be tested for STIs (10). Routine NAAT testing for women with vaginal complaints provides the opportunity for increased accuracy in diagnosis and treatment for both the underlying cause of vaginal symptoms and for the presence of STIs. A possible scenario for risk-based screening for STIs using NAATs for the diagnosis of vaginitis instead of Gram stain and culture could entail reflex STI NAAT testing in the laboratory for women who are NAAT-positive for BV. This approach has the potential to positively impact STI control; however, implementation of such a scheme is dependent on outcomes of investigations into the accuracy of identifying the risk of concomitant STI by relying on a NAAT-based diagnosis for the underlying cause of vaginal inflammation. Whether MG should be included along with CT, NG, and TV in such an algorithm will depend on results from additional future studies designed to understand the longitudinal outcomes associated with untreated MG infection in women with vaginitis.

A strength of this study is the assessment of specimens collected prospectively from a large cohort of women enrolled from 21 geographic sites and a variety of clinical practice types across the United States. Also important are the employment of consensus Nugent score and standardized methodology for obtaining Amsel criteria results for BV diagnosis, the use of dual media culture for diagnosis of candidiasis, and the use of highly sensitive FDA-cleared NAATs for STI diagnosis.

Limitations of the study include lack of comparative analyses for the 317 women in the cohort with signs and symptoms of vaginitis/vaginosis, but who were negative for BV, candidiasis, and all four STIs; some of the women enrolled in this category were originally diagnosed with non-infectious causes of irritation/itching/burning such as desquamative inflammatory vaginitis, irritant dermatitis, and lichen sclerosus. We also did not include HIV infection status in the analysis of the women enrolled in the study although most (99%) women in the cohort were categorized as HIV-negative. Finally, the number of CT and NG infections in the cohort studied was relatively small which may have led to inaccuracies in estimating the significance of association of these STIs with clinical and laboratory diagnoses. In spite of this, we did find that, similar to previous studies, women with only CT infections had an OR of 2.44 for infection in BV+ vs BV− (adjusting for VVC) although this increase in risk was not significantly different from controls.

In conclusion, we found high rates for sexually transmitted infections in women seeking care for symptoms of vaginitis and bacterial vaginosis, revealing highly complex associations of STIs with two of the major causes of vaginal dysbiosis. *Trichomonas vaginalis* and *Mycoplasma genitalium* were significantly associated with bacterial vaginosis independent of candidiasis and other STI infections. These results underscore the importance of STI testing in women seeking care for symptoms of vaginal inflammation and abnormal discharge.
